# Performance assessment in elite table tennis matches using the enhanced first offensive shot model

**DOI:** 10.1186/s13102-025-01183-6

**Published:** 2025-06-03

**Authors:** Ruizhi Liu, Frederic Rothe, Martin Lames

**Affiliations:** 1 China Table Tennis College, Shanghai University of Sport, 650 Qingyuanhuan Road, Shanghai, 200438 China; 2 School of Medicine and Health, Technical University of Munich, Georg-Brauchle-Ring 60/62, 80992 Munich, Germany

**Keywords:** Athletic performance, State transition, Gender difference, Observational analysis, Racket sports, Olympic Games Tokyo 2020

## Abstract

**Background:**

Technique and tactics are key elements in assessing table tennis performance and have been widely studied. However, most existing methods classify shots solely by sequence or shot number, overlooking the tactical attributes and their impact on rally outcomes. The First Offensive Shot (FOS) model attempted to address this issue but remained too coarse-grained. To overcome these limitations, this study introduces the Enhanced FOS model (EFOS), which integrates shot type and shot number into a more refined state-transition framework. This novel approach provides a more detailed representation of rally dynamics, enabling a deeper analysis of rally progression.

**Methods:**

The EFOS model divides a rally into three phases: the pre-offensive phase (POP), the initial-offensive phase (IOP), and the final-offensive phase (FOP). Each phase is further categorised based on shot type and shot number. Transition probabilities between states were computed. A comprehensive analysis was conducted on 105 men’s and women’s singles matches from the Olympic Games Tokyo 2020, with gender, ranking, and match results as independent variables.

**Results:**

The IOP accounts for the highest usage (70.6%) among the three phases, with FOS and the following shot–FOS + 1–rank second. Male players tend to use defensive techniques to return shots and attack later, whereas female players prefer to attack defensive shots directly. High-ranked players (≤ 40) demonstrated superior transition efficiency and lower error rates, particularly in the IOP, while winners consistently exhibited higher transition rates and fewer errors across all phases.

**Conclusions:**

Firstly, the initial-offensive phase emerges as pivotal, with the first offensive shot and the subsequent shot significantly influencing rally outcomes. Secondly, attacking defensive shots have proven to be more effective than attacking serves. Thirdly, male players outperform female players in both initial and subsequent attack. These findings offer practical insights for players and coaches, suggesting that training should emphasise the initial-offensive phase and focus on reducing errors during transitions, particularly for low-ranked players. Additionally, gender-specific training strategies may be developed to address the observed performance differences.

## Introduction

In performance analysis, a discipline of sports science, game sports are seen as dynamic interaction processes with emergent behaviour [1]. The complexity of techniques and tactics, along with their flexible application in a match, is a critical factor in game sports [2]. This concept has long been widely accepted within the field of table tennis [3–6]. As a consequence, technical and tactical analysis are key elements in the analysis of table tennis performance, accounting for a large proportion of the published research.

Methods of technical and tactical analysis in table tennis originate from simple mathematical statistics by Wu Huanqun [7]. His pioneering three-phase evaluation method [8] has significantly shaped the domain of technical and tactical analysis in table tennis, especially in Asia. Other researchers have further enriched and expanded this methodology. These expansions include studies on technical effectiveness and competition performance [9], four-phase evaluation theory [10], double five sections analysis [11], new shot number-based approach [12], dynamic three-phase method [13], modified Wu Huanqun’s method [14], and other notational analysis methods combining shot number, technique, placement, and footwork [15, 16]. In sum, performance-related variables in game sports, so-called performance indicators, have been a key issue in performance analysis in table tennis [17].

All these well-known approaches have in common that they classify shots by their sequence, i.e., the number of the shot in the rally. Applying the concept of equivalence classes [1] to shot classifications presupposes a high similarity of shots within assigned shot classes. On the other hand, different shot classes should exhibit a sufficient degree of dissimilarity. Using classifications based on shot number may lead to inconsistent shot classes by incorporating a variety of different shot types. For example, the third shot in table tennis can vary from a short, defensive backspin to an above-table offensive topspin or even an all-in topspin attack from behind the table. Thus, general statistics concerning the specific shot number can be insufficient for describing technical or tactical constructs.

To address this problem, Fuchs and Lames [18] introduced a new method for categorising shots in table tennis based on the first offensive shot (FOS). They modelled a rally in table tennis as a sequence of service, defensive shots, the FOS, and finally offensive shots. Within this categorical system, each shot can naturally result in a subsequent shot of the opponent, an error, or a point. Individual shots are assigned to the aforementioned shot classes that are tactically and technically similar. This also underlines the role of the FOS as a “turntable” of the rally.

A similar approach was introduced by Yu and Gao [4], who presented a three-phase model, which is very similar in concept to the FOS. They separated table tennis rallies into a mutual restriction phase, an initial attack phase, a counterattack phase, and a topspin exchange phase according to the type of technique utilised. Both groups studied the detailed technical utilisation characteristics and winning rates.

Nevertheless, the models suggested by these two studies suffer from some common shortcomings. First, it can make a large difference whether the FOS attacks the serve (second shot) or a defensive shot (third and ≥ fourth shot). The serve, which is the only unrestricted technique in table tennis, is executed without being preceded by any other shot; therefore, it is more intricate than a defensive shot. Consequently, attacking the serve with a FOS arguably involves a greater risk than playing a defensive shot. Second, it is questionable whether all the shots after the FOS should fall into the equivalence class of offensive shots. Since the FOS is inherently challenging and most serves are dropped closer to the central part of the net, the usage rate of some receiving techniques, such as forehand or backhand flip has increased substantially in recent years [16]. These techniques are executed above, instead of behind the table, making them less suitable for an open, topspin approach. Hence, it can be assumed that one may not be able to exhibit as much pressure with a FOS compared to attack shots later in the rally. On the other hand, shots after the FOS are impacted by the quality and outcome of the FOS and show, for example, a different winner-error profile compared to shots in the final topspin-exchange phase.

The importance of an offensive playing style and shots, or first offensive shots in various contexts has been well-studied by many scholars [5, 19, 20] and therefore will not be detailed here. Although the FOS model [18] provided fundamental knowledge about FOS, its structure was overly simplistic, and its findings were limited to descriptive statistics, making it inadequate for capturing the dynamic nature of the play. To overcome these limitations, this study introduces the Enhanced First Offensive Shot model, a novel framework that incorporates a more differentiated state-transition structure by integrating shot type and shot number. This enhanced model enables a more detailed representation of within-rally dynamics. The transition states in this model serve as a new family of performance indicators, offering deeper insights into tactical patterns and rally progression in table tennis. Using a representative sample of world-class matches from the Olympic Games Tokyo 2020 (OG20), the following hypotheses were tested:

1. The initial-offensive phase is the most important phase.
2. Attacking defensive shots is more effective to score than attacking serves.
3. The performance of male players under the EFOS model is different from that of female players.

## Methods

This study applied an observational design that was nomothetic, follow-up, and multidimensional (N/F/M) [21]. It enabled a systematic analysis of the behavioural patterns exhibited by elite table tennis players during matches. The nomothetic aspect involved the analysis of multiple players to identify generalisable FOS transition features. The follow-up component comprised both inter-session and intra-session observations, allowing for a comprehensive analysis of different populations across multiple matches and within individual matches, respectively. The multidimensionality of the design is reflected in the analysis of different technical and tactical dimensions such as the rally phases and shot numbers. The study employed non-participant observation as all the data were collected via video observation after the match. All the players competed in a natural environment, i.e. in the field of play, under the jury of match officials, during the real table tennis match.

### Participants

A total of 105 men’s and women’s singles matches (best of 7 sets) from the OG20 (Table 1) were included in the analysis. All included players were of the offensive playing style, as indicated in the official player profiles published on the International Table Tennis Federation (ITTF) website, to minimise bias in the FOS statistics arising from participant variability. This selection ensures consistency in data interpretation, as defensive players exhibit substantially different transition patterns that do not align with the concept of EFOS. Ranking positions were obtained from the Men’s and Women’s Singles ranking list of July 20th, 2021, prior to the Olympic Games provided by the ITTF.

**Table 1:** Basic information of the participants

Gender	No. of Players	No. of nationalities	Ranking range	No. of Matches	No. of sets	No. of Rallies
M	60	40	1st − 383rd	56	302	5,505
F	56	36	1st − 511th	49	248	4,420
Total	116	51	1st − 511th	105	650	9,925

Note: M, for male; and F, for female

### Resources

#### Observation instrument

This study applied an ad hoc observation instrument specifically developed for the study of FOS, all the criteria were the mandatory elements for the independent variables or EFOS model. And its validity has already been empirically demonstrated [18]. The instrument comprises a six-category system, with each category defined by specific field formats (Table 2).

**Table 2:** Criteria of the observation instrument

No.	Criteria	Description	Categories and codes
1	Gender	The observed gender of the participant as recorded for the tournaments	• M (male) • F (female)
2	Server	The player who serves first in a scoring rally	• First (the player whose name is listed first when entering match information in the analysis system). • Second (the player whose name is listed second when entering match information in the analysis system)
3	Rally length	The sum of legal shots in a scoring rally	• 1 (one shot in total in this rally) • 2 (two shots in total in this rally) •… • x (x shots in total in this rally)
4	FOS number	The shot number at which the FOS occurs in a scoring rally	• 0 (no FOS in the rally) • 2 (the second shot) •… • x (the xth shot)
5	FOS player	The player who first launches the offensive shot in a scoring rally	• No (no FOS in this rally) • First (same with above) • Second (same with above)
6	Winner	The player who scores in the rally	• First (same with above) • Second (same with above)

#### Recording instrument

All the singles matches of the OG20 were recorded using private video recordings of the television program Eurosport Germany. Participants of the OG20 agreed “to be filmed, televised, photographed, identified and otherwise recorded at the Games” and for the additional usage [22]. All the data from matches involving non-defensive-players were collected using the table tennis match analysis software TUM.TT [23]. 25 rallies were excluded during the data collection since the video footage prevented accurate identification of shot types. No ambiguous cases were recorded in terms of FOS classification, as the distinction between offensive and defensive techniques in the EFOS model is based on clear observable criteria. The collected data were then exported to Microsoft Excel 365 for the next step. SPSS 25.0 was used for further statistical analysis.

### Reliability of the data

All data collection was carried out by an undergraduate student, who is also a German B-licensed table tennis coach working at a Bundesliga club. Prior to data collection, the student received formal training on the use of the TUM.TT tool and the EFOS model coding protocol to ensure consistency. The training consisted of two main stages. First, the observer engaged in self-directed learning of the observation instrument, including the six coding criteria and their corresponding categories and codes. In the second stage, the observer conducted joint coding sessions with a senior expert—an experienced table tennis coach from the German Paralympic Committee as well as the developer of the TUM.TT software. They coded two full matches (one men’s and one women’s singles match) together. During this process, the two coders reached full agreement on all variables, including rally length, FOS number, and FOS player. These joint sessions ensured a shared understanding of the EFOS coding framework and reduced the likelihood of subjective misinterpretation.

The inter-rater reliability test was performed by a table tennis performance analyst with over ten years of experience working with a national table tennis team, who also underwent the same training to standardise coding decisions. The test was conducted using the kappa statistic on a randomly selected sample of four 7-set matches (2 men’s and 2 women’s matches), comprising 529 rallies. The kappa coefficients for the six indicators (gender, server, rally length, FOS number, FOS player and winner) were 1.000, 1.000, 0.972, 0.988, 0.998, and 1.000, respectively. These coefficients were rated as “very good” [24]. Given the high inter-rater reliability results, intra-rater reliability testing was deemed unnecessary [1]. Although intra-rater reliability testing was not conducted, routine consistency checks were implemented throughout the data collection process. These included periodic coding reviews and recalibration sessions based on the EFOS protocol. Given the coder’s formal training, domain expertise, and the excellent inter-rater reliability results, the likelihood of intra-rater drift was considered minimal.

### Enhanced first offensive shot model

In line with the conceptual foundations of the EFOS model, transition probabilities were prioritized as the primary performance indicators. Unlike isolated measures such as rally length or shot number, which offer only descriptive insights, transition probabilities capture the sequential nature and tactical flow of rallies. They represent the likelihood of a player’s shot leading to specific subsequent outcomes (e.g., continued rally, point, or error), thereby reflecting decision-making efficacy and consistency under pressure. This modeling choice follows established practices in state-transition-based performance analysis, particularly in fast-paced net games such as table tennis [1, 25].

**Fig. 1:**
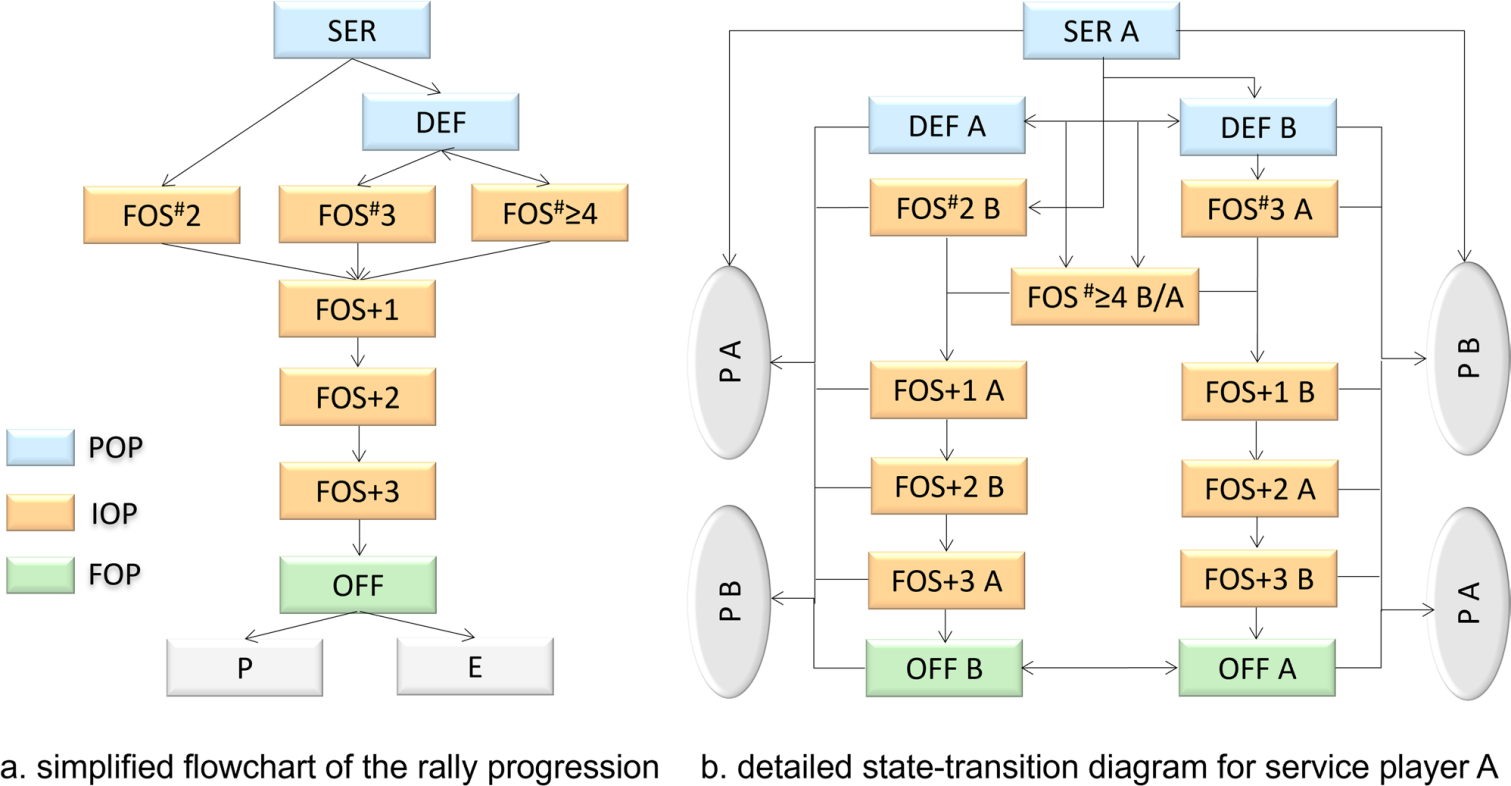
Overview of the Enhanced First Offensive Shot (EFOS) model. Legend: SER denotes service; DEF denotes defensive shot. OFF denotes offensive shot. P or E stands for point or error, respectively. “A” or “B” appended at the end of each frame denotes which player executed the shot

In accordance with the original definition [18], the first offensive shot in a rally is defined as the first shot following the serve without any kind of backspin/side backspin. Building upon this definition, the authors of this study propose an enhanced model by dividing the EFOS model into three phases: the pre-offensive phase (POP), the initial-offensive phase (IOP), and the final-offensive phase (FOP) (Fig. 1). Each phase is further categorised.

Each rally begins with the serve, constituting the first shot in the rally. Subsequently, there can be between 0 to n shots before an offensive shot is executed. This incipient phase is termed the pre-offensive phase. Once an offensive shot is made, the rally progresses into the initial-offensive phase. The FOS itself is differentiated into three types/states: FOS^#^2 (the second shot in a rally attacking the serve), FOS^#^3 (the third shot in a rally attacking the first defensive shot from the receiving player), and FOS^#^≥4 (all subsequent FOSs). Expanding the original categorisation of FOS [18], we introduce the next three consecutive shots, referred to as FOS + 1, FOS + 2 and FOS + 3, as distinct states. This expansion is motivated by the findings of prior study [18]. Empirically, different success and error rates compared to typical attack shots can be observed until FOS + 3 (Figs. 3 and 4; Table 4). All subsequent shots are collectively fall under the category of offensive shots in the final-offensive phase. Points and errors signify the absorption states of a rally and can occur in any state.

A state-transition model encompasses all possible states within a system such as the rallies in one or more table tennis matches, along with the structural and empirical transitions between these states. The structural transitions, as elucidated by the EFOS model above, are illustrated in Fig. 1. Figure 1a outlines the sequential development from the serve to the final outcome under the framework of EFOS model. Figure 1b illustrates every possible transition between POP, IOP, FOP states and the absorbing outcomes from the view of service player A. Their empirical representation is presented in the form of a transition matrix, detailing the transitions from each state (rows) to any other state (columns). The empirical transition matrix for all matches in the sample is given in Table 4.

To describe a table tennis match, each shot is treated as an observational unit and categorised into different states based on the type of technique and shot number after the FOS. This classification follows the structure outlined in Fig. 1, where each shot category represents a unique state. The colour scheme in the flow chart illustrates the different phases of the rally (POP, IOP & FOP).

The serve constitutes the starting state in the state-transition model (assigned to player A in Fig. 1, the transition model for service player B can be obtained with appropriate changes in denomination of states). Transient sets of states are states where the rally may stay in, but once this set is left, there is no return. There are two transient sets of states in the model depicted in Fig. 1b: defensive A and defensive B as well as offensive A and offensive B. Transient states, i.e., states where the rally may only enter once and then leave without option of return, are FOS^#^2, FOS^#^3, FOS^#^≥4, FOS + 1, FOS + 2 and FOS + 3. Finally, we have the absorbing states Point A and Point B. Seen from a modelling standpoint, this structure represents the efforts of the two players to reach their goal (Point A or Point B). The interactive nature is captured by the level of the corresponding transition probabilities, which can be seen as the result of this antagonistic struggle.

Transition probabilities between states were computed empirically as the proportion of shots transitioning from one state to another. Specifically, for any two states X and Y, the transition probability P_(X → Y)_ was calculated as:

1 $$\eqalign{& {P_{(X \to \>Y)}} \cr & = {\matrix{ Number\>of\>transitions\> \hfill \cr from\>state\>X\>to\>Y \hfill \cr} \over \matrix{ Total\>number\>of\> \hfill \cr shots\>originating\>from\>state\>X \hfill \cr} } \cr} $$

### Independent variables

Gender, ranking, and match result were included as independent variables in the study. The world ranking of 40th place was set as the division point to minimise the difference in the number of matches between groups with respect to gender, thereby reducing potential statistical errors arising from sample sizes (Table 3).

**Table 3:** Distribution of the independent variables of gender, ranking and match results

Category		M		F		Total	
		Count	%	Count	%	Count	%
Ranking	≤ 40	57	27.1	40	19.1	97	46.2
	> 40	55	26.2	58	27.6	113	53.8
Match result	W	56	26.7	49	23.3	105	50.0
	L	56	26.7	49	23.3	105	50.0

Note: ≤40 or > 40 indicates that the international ranking is within or outside the top 40, respectively. W or L stands for win or loss, respectively

### Data analysis

All computations for each transition state in the EFOS model, as well as for Fig. 3 (Formula 2,3, and 4), Fig. 5 (Formula 5 and 6), and Fig. 5 (Formula 7), were executed in Excel.

2 $$\eqalign{& Direct\>winning\>rate\>of\>FO{S^\# }i \cr & = \>{{Count\>of\>winning\>shots\>of\>FO{S^\# }i} \over {Count\>of\>FO{S^\# }i\>}} \cr} $$

3 $$\eqalign{& Overall\>winning\>rate\>of\>FO{S^\# }i \cr & = \>{\matrix{ Count\>of\>winning\>shots\>of\> \hfill \cr FO{S^\# }i,FO{S^\# }i + 1, \hfill \cr FO{S^\# }i + 2,FO{S^\# }i + 3\> \hfill \cr} \over {Count\>of\>FO{S^\# }i\>}}\>\> \cr} $$

4 $$\eqalign{& Usage\>Rate\>of\>FO{S^\# }i \cr & = \>{{Count\>of\>FO{S^\# }i} \over {Count\>of\>sample\>rallies\>( = \>9925)\>}} \cr} $$

5 $$\eqalign{& Direct\>winning\>rate\>of \cr & \>the\>corresponding\>FOS - stat{e^\# }i \cr & = \>{\matrix{ Count\>of\>winning\>shots\>on\> \hfill \cr the\>corresponding\>FOS - stat{e^\# }i\> \hfill \cr} \over \matrix{ Count\>of\>corresponding\> \hfill \cr FOS - stat{e^\# }i \hfill \cr} } \cr} $$

6 $$\eqalign{& Usage\>rate\>of\>the \cr & corresponding\>FOS - stat{e^\# }i \cr & = \>{\matrix{ Count\>of\>corresponding\> \hfill \cr FOS - stat{e^\# }i \hfill \cr} \over {Count\>of\>sample\>rallies\>( = \>9925)\>}} \cr} $$

7 $$\eqalign{& Winning\>rate\>of\>FO{S^\# }i\>and\>FO{S^\# }i + 2 \cr & = \>{\matrix{ Count\>of\>winning\>shots\> \hfill \cr of\>FO{S^\# }i\>and\>FO{S^\# }i + 2 \hfill \cr} \over {Count\>of\>FO{S^\# }i\>and\>FO{S^\# }i + 2}} \cr} $$

In all above formulae, i stands for the FOS categories of 2, 3, or ≥ 4.

Descriptive statistics were calculated using the mean for each index. The Kolmogorov‒Smirnov test indicated that 24 out of 30 transition probabilities were not normally distributed, thus, nonparametric tests were uniformly applied for consistency. The Mann‒Whitney U test (*p* < 0.05) was employed to test differences based on gender, ranking, and match results. The Kruskal‒Wallis H test (*p* < 0.05) was applied to assess differences across different FOS categories in the IOP. Pairwise comparisons were conducted using the Holm–Bonferroni method. All the statistical analyses were carried out using SPSS 25.0.

## Results

### EFOS model

**Fig. 2:**
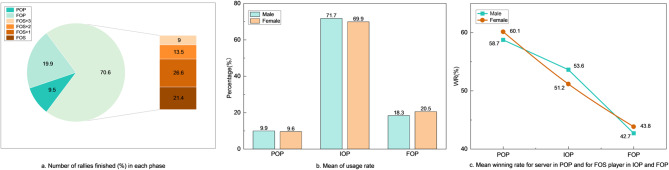
Descriptive results for the EFOS model of the match sample

Figure 2a illustrates the distribution of rallies finished in the POP, IOP, and FOP, accounting for 9.5%, 70.6% and 19.9% of the 9,925 rallies, respectively. Notably, the IOP accounted for the majority of rallies, highlighting its critical role in determining outcomes. Within the IOP, the proportions of FOS, FOS + 1, FOS + 2, and FOS + 3 were 21.4%, 26.5%, 13.5%, and 9.0%, respectively, indicating that many outcomes are determined within the first two shot of the IOP, i.e. FOS and FOS + 1. Gender comparisons revealed no significant differences in phase usage or winning rates (Fig. 2b and c), suggesting similar strategic patterns between male and female players in phase utilisation.

### General transition matrix

Table 4 displays the average transition probabilities between states s in the 105 matches. Nearly half of the rallies involved an immediate attack on the serve, with 45.3% of services transitioning from POP to IOP via FOS^#^2. The remaining services (52.5%), which were successfully pushed by receivers, were most likely attacked by servers on the third shot (42.8%). When transitioning to the IOP, the transition probabilities from FOS^#^2, FOS^#^3, and FOS^#^≥4 to FOS + 1 were similar. FOS + 1, FOS + 2 and FOS + 3 occupied the top three highest direct error rates across all states. During the FOP, transition probabilities from OFF to OFF, P and, E were similar to those in the last two episodes of the IOP. Notably, players’ direct error rates were multi-fold greater than their direct winning rates across all conditions, highlighting the challenge of maintaining consistency during transitions.

**Table 4:** Transition matrix (%) for all matches (*N* = 105) and rallies (*N* = 9925)

	DEF	FOS^#^2	FOS^#^3	FOS^#^≥4	FOS + 1	FOS + 2	FOS + 3	OFF	*P*	E
SER	52.5	45.3							0.7	1.4
DEF	28.9		42.8	18.3					1.6	8.4
FOS^#^2					77.2				3.7	19.1
FOS^#^3					76.1				6.6	17.3
FOS^#^≥4					73.8				7.4	18.9
FOS + 1						61.5			5.4	33.1
FOS + 2							68.2		4.7	27.1
FOS + 3								68.8	5.4	25.8
OFF								69.9	4.3	25.7

Note: SER denotes service; DEF denotes defensive shot. OFF denotes offensive shot. P or E stands for point or error, respectively

### Independent variables

Table 5 presents the results of the statistical tests for comparisons between genders, rankings, and results. Male players showed significantly higher transition probabilities in DEF to DEF (25.4% vs. 20.5%, *p* = 0.004) and preferred later FOS^#^≥4 attacks (21.0% vs. 16.3%, *p* = 0.001). Female players favoured earlier FOS^#^3 attacks (51.7% vs. 42.6%, *p* < 0.001). The significantly higher transition possibilities from FOS^#^≥4, FOS + 2, and OFF to P may reflect gender-specific tactical characteristics, such as differences in risk tolerance or technique quality.

**Table 5:** The Mann‒Whitney U test on transition probabilities (%) between genders (N_M_ = 112, N_F_ = 98), rankings (N_≤ 40_ = 97, N_> 40_ = 113) and results (N_W_ = 105, N_L_= 105)

Transitions		Gender				Ranking				Result			
From	To	Mean_M_	Mean_F_	*p*	*r*	Mean_≤ 40_	Mean_> 40_	*p*	*r*	Mean_W_	Mean_L_	*p*	*r*
SER	DEF	52.5	52.3	0.961	< 0.01	52.7	52.2	0.807	0.02	52.0	52.8	0.696	0.03
	FOS^#^2	45.1	45.6	0.984	< 0.01	45.4	45.2	0.965	< 0.01	45.9	44.8	0.631	0.03
	P	0.8	0.8	0.986	< 0.01	0.7	0.9	0.578	0.04	1.0	0.6	0.132	0.10
	E	1.6	1.3	0.251	0.08	1.2	1.8	0.273	0.08	1.2	1.7	0.294	0.07
DEF	DEF	25.4	20.5	**0.004**	0.20	24.9	21.5	**0.035**	0.15	23.0	23.1	0.771	0.02
	FOS^#^3	42.6	51.7	**< 0.001**	0.31	45.8	47.8	0.383	0.06	47.8	46.0	0.427	0.06
	FOS^#^≥4	21.0	16.3	**0.001**	0.23	19.9	17.8	0.159	0.10	18.8	18.7	0.947	0.01
	P	1.7	2.3	0.056	0.13	1.7	2.2	0.660	0.03	2.7	1.2	**< 0.001**	0.24
	E	9.6	9.5	0.730	0.02	7.9	11.0	**< 0.001**	0.24	7.9	11.2	**< 0.001**	0.25
FOS^#^2	FOS + 1	76.0	77.4	0.516	0.05	79.2	74.5	**0.015**	0.17	78.8	74.5	**0.027**	0.15
	P	4.5	3.3	0.062	0.13	3.5	4.3	0.564	0.04	4.0	3.9	0.83	0.02
	E	19.5	19.3	0.977	< 0.01	17.3	21.2	**0.011**	0.18	17.2	21.6	**0.012**	0.17
FOS^#^3	FOS + 1	74.5	74.9	0.965	< 0.01	77.6	72.1	**0.005**	0.19	78.5	70.8	**< 0.001**	0.25
	P	7.5	6.4	0.250	0.08	7.2	6.8	0.656	0.03	7.5	6.4	0.500	0.05
	E	18.1	18.8	0.715	0.03	15.2	21.1	**0.003**	0.20	14.0	22.8	**< 0.001**	0.32
FOS^#^≥4	FOS + 1	69.4	71.2	0.095	0.12	71.7	69.0	0.953	< 0.01	78.4	62.1	**< 0.001**	0.32
	P	8.9	5.8	**0.001**	0.23	9.8	5.4	**0.007**	0.19	8.1	6.8	0.988	< 0.01
	E	19.1	19.0	0.103	0.11	17.5	20.3	0.463	0.05	13.5	24.5	**< 0.001**	0.25
FOS + 1	FOS + 2	58.2	61.7	0.090	0.12	60.7	59.1	0.452	0.05	56.8	62.9	**< 0.001**	0.23
	P	6.1	5.5	0.336	0.11	5.8	5.8	0.419	0.04	4.5	7.2	**0.002**	0.35
	E	35.7	32.8	0.103	0.07	33.5	35.1	0.527	0.06	38.7	30.0	**< 0.001**	0.21
FOS + 2	FOS + 3	67.1	66.7	0.974	< 0.01	69.0	65.2	0.095	0.12	70.0	63.9	**< 0.001**	0.24
	P	6.2	4.0	**0.045**	0.14	6.0	4.4	0.117	0.11	6.1	4.2	**0.026**	0.15
	E	26.7	29.4	0.324	0.07	25.1	30.4	**0.009**	0.18	23.9	32.0	**< 0.001**	0.31
FOS + 3	OFF	67.1	67.5	0.838	0.01	68.1	66.6	0.375	0.06	64.0	70.6	**0.005**	0.20
	P	5.6	4.6	0.103	0.02	5.9	4.6	0.128	0.08	4.3	6.1	**0.014**	0.27
	E	27.3	27.9	0.833	0.11	26.1	28.8	0.245	0.11	31.8	23.4	**< 0.001**	0.17
OFF	OFF	65.9	66.1	0.969	< 0.01	66.9	65.2	0.381	0.06	69.5	62.5	**0.009**	0.18
	P	5.4	3.5	**< 0.001**	0.26	5.5	3.7	**0.005**	0.19	5.4	3.7	0.108	0.11
	E	28.7	30.4	0.612	0.04	27.6	31.1	0.119	0.11	25.1	33.9	**< 0.001**	0.25

Note: Effect size (r) interpretation: Small ≥ 0.1, Medium ≥ 0.3, Large ≥ 0.5

High-ranked players (≤ 40) displayed higher transition rates and lower direct error rates when progressing from defensive play (DEF to DEF), initiating an attack (FOS^#^2 to FOS + 1; FOS^#^3 to FOS + 1) and counterattacking (FOS + 1 to FOS + 2). Low-ranked players exhibited higher error rates in these transitions, highlighting the superior attack efficiency and consistency of elite players. Winners maintained higher transition rates and lower direct error rates across all offensive states (IOP and FOP).

### Initial-offensive phase

**Fig. 3:**
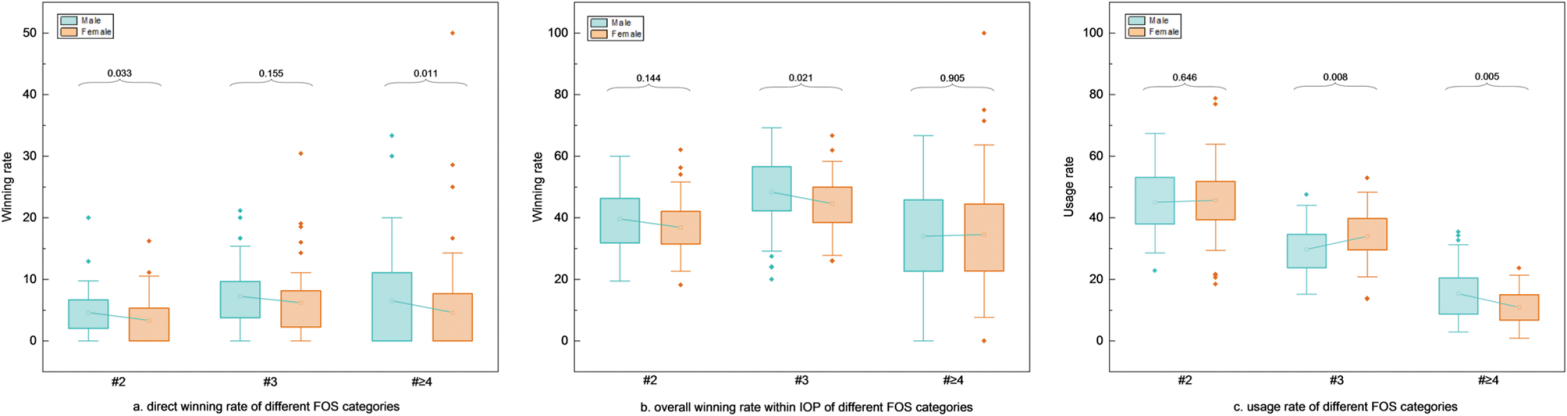
Comparison of direct winning rates on FOS, winning rates within IOP, and usage rates in each FOS category between gender. Legend: The bottom and top edges of the box and whiskers represent the first quartile, third quartile, minimum and maximum, respectively.  represents the mean. ♦ represents outliers, which are values beyond 1.5 times the interquartile range from the nearest quartile. The value above the bracket represents the *p* value between each pair

A more in-depth analysis of the rallies in the initial-offensive phase (excluding the rallies ending in the POP and FOP) revealed that the second shot had the highest FOS frequency, accounting for 45.2% and 45.4% for male and female players, respectively (Fig. 3c). It was followed by the third shot (M: 29.7%; F: 34.1%) and the ≥ fourth shot (M: 15.5%; F: 11.1%). Significant differences in usage rates were found in the third (*Z* = − 2.646, *p* = 0.008) and ≥ fourth (*Z* = 2.833, *p* = 0.005) FOS categories. Figure 3a and b depict the direct winning rates for FOS in different FOS categories, along with the winning rates for the entire IOP (FOS, FOS + 1, FOS + 2, FOS + 3). Male players had higher direct winning rates than female players in all categories, with statistical significance only in the second (*Z* = 2.138, *p* = 0.033) and ≥ fourth (*Z* = 2.555, *p* = 0.011) categories. In contrast, male players appeared to have significantly higher winning rates than females only in the three consecutive shots following the third shot (*Z* = 2.316, *p* = 0.021).

**Fig. 4:**
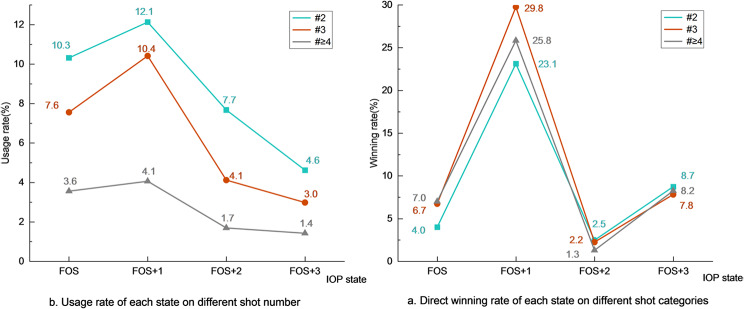
Direct winning rate and usage rate of FOS players in different FOS categories within the IOP

In terms of the frequency of each state in the IOP, FOS + 1 had the highest usage rate, regardless of the shot category in which FOS occurred (Fig. 4b). FOS followed in second place, followed by FOS + 2 and FOS + 3. Regarding the direct winning rate of each state (Fig. 4a), the order from highest to lowest was FOS + 1, FOS + 3, FOS, and FOS + 2.

Significant differences in the direct winning rates were observed among the different shot categories, particularly for FOS, FOS + 2 and FOS + 3, suggesting varying levels of effectiveness across these states (Table 6). The direct error rates were observed only for FOS + 1, indicating a potential vulnerability in this state.

**Table 6:** The Kruskal‒Wallis H test among different FOS occurrence (%) shot categories (*N* = 105 for each category)

Transition		Mean_#2_	Mean_#3_	Mean_#≥4_	*p*	*ε* ^*2*^
From	To					
FOS	FOS + 1	76.6	75.3	72.7	0.302	0.00
	P	4.0	6.7*	7.0	**0.002**	**0.02**
	E	19.4	18.0	20.3	0.566	0.04
FOS + 1	FOS + 2	63.8*	54.7	57.8	**< 0.001**	**0.06**
	P	5.5	5.4	7.1	0.126	0.02
	E	30.7	39.9*	35.2	**< 0.001**	0.03
FOS + 2	FOS + 3	66.4	67.3	68.4	0.084	0.00
	P	5.4	5.7	4.00*	**< 0.001**	**0.11**
	E	28.2	27.0	27.6	0.134	0.09
FOS + 3	OFF	67.3	67.3	63.4	0.865	0.03
	P	5.2	4.9	5.6*	**0.023**	**0.05**
	E	27.5	27.9	31.1	0.875	0.09

Note: * indicates that a significant difference was observed between this value and the other two, according to the pairwise comparisons (FOS + 3 – P of Mean_#≥4_ only different from Mean_#2_). Effect size (ε²) interpretation: No < 0.01, Small ≥ 0.01, Medium ≥ 0.06, Large ≥ 0.14

## Discussion

This study introduces the Enhanced First Offensive Shot (EFOS) model, extending the framework of FOS to provide a detailed state-transition model for understanding table tennis interactive dynamics. By categorising rallies into three distinct phases—pre-offensive, initial-offensive, and final-offensive—the EFOS model offers a comprehensive perspective on the technical attributes and tactics employed by players during matches. In particular, the focus on differentiating the initial-offensive phase, i.e., the introduction of FOS-states according to shot number (FOS^#^2, FOS^#^3, FOS^#^≥4) and post-FOS states (FOS + 1, FOS + 2, FOS + 3), allows for a deeper investigation of shot sequences and their impact on rally outcomes.

The overall transition probabilities observed in the EFOS model reveal an interesting pattern: the the direct winning rate was lower than the direct error rate across all states, indicating that most points were won through opponent errors. This result is consistent with the findings in table tennis [6], badminton [26], tennis [25], and seems to constitute a general law in single net games. Hence, to precisely assess the scoring advantage of FOS, it is necessary to take into account the outcomes of a minimum of two successive shots, constituting one exchange. As indicated in the previous study [18], the accumulated percentage of rallies ending at FOS (21.0%), FOS + 1 (44.3%), FOS + 2 (62.9%), and FOS + 3 (73.9%) experiences a rapid increase with significant intervals, followed by a gradual decline from FOS + 4 onwards. Thus, within the EFOS, the IOP incorporated FOS and the subsequent three shots.

Each rally started from POP, but only 9.5% of the 9,925 rallies finished in this phase in OG20; thus, 90.5% of the rallies contained a FOS. This proportion is similar to the data obtained for OG16 (9.0% and 90.9%, N_match_ = 90, N_rally_ = 7,449) [18]. This implies that there were no big changes in the last two Olympic cycles. The figures in Yu and Gao [4] are slightly different (7.1% and 93.6%, N_match_ = 56, N_rally_ = 5,507), potentially resulting from their sample focusing on final-stage matches in top-level tournaments. Slight variations are found in the proportions of IOP and POP. In OG20, these two phases accounted for 70.6% and 19.9%, respectively, compared to 67.1% and 23.8%, respectively, in OG16. The observed disparity might indicate an increased importance of IOP in OG20.

From Tables 4 and 6, it is evident that the direct winning rates of FOS^#^3 and FOS^#^≥4 were significantly greater than those of FOS^#^2. FOS + 1, the shot counterattacked by the opponents of FOS players, FOS^#^3 + 1 and FOS^#^≥4 + 1, showed higher direct error rates and lower transition rates to the next state than did FOS^#^2 + 1. For FOS + 2 and FOS + 3, there was no pronounced advantage when launching an attack on the second shot compared to the third, fourth and subsequent shots. Taken together with Figs. 3 and 4, it can be deduced that the scoring efficacy of FOS^#^3 and FOS^#^≥4 is evidently greater than that of FOS^#^2. In other words, attacking defensive shots is more efficient than attacking serves. This is easily understandable, as a quality serve poses a significant threat to the receiver [27], and the transition probability from serve to point has increased over the past decade [28]. As the number of shots increases, the advantage conferred by the serve to the server diminishes, while the success rate of the player’s FOS gradually increases, thereby providing an explanation or reinforcement for this result.

There were similar proportions of male and female players using either defensive or offensive techniques when returning serves, whereas disparities were found when dealing with defensive shots (Table 5). Female players attacked more frequently (9.1%) on the third shot, while male players adopted defensive techniques more often (5.0%) to return defensive shots and attack later. A detailed shot-by-shot technical analysis of 347 top-level matches by Zhou and Zhang [29] concluded that the most effective tactics in the receiving round in men’s matches involved returning a short serve with a short touch to force the opponent into a touch short or push in the third shot. The player then attacked the ball in the fourth shot, achieving a high scoring rate in the rally. In contrast, female players preferred pushing the ball to the endline area on the second shot but not with a touch shot. They opted for their opponents to initiate the first offensive shots and counterattack the topspin. These transition probabilities coincidentally verified the winning patterns summarised by these two scholars.

From the perspective of ranking and match results (Table 5), winners and losers, high-ranked and low-ranked players, differed significantly in many transitions, as expected [1]. Both winners and high-ranked players exhibited superior abilities in transitioning to the next state, higher direct winning rates, and lower error rates in almost all states in the POP, IOP, and FOP, except for the serve, where no statistical differences were observed among players in any of the independent variables. The serve is independently executed by a player and is not influenced by the opponent. Therefore, differences among players of varying performance levels were not significant for all serve transitions [30]. The aforementioned observation fully demonstrates that elite players are characterised by a high success rate, highlighting their superior performance. Furthermore, superior players exhibit more pronounced disparities in both IOP and FOP than low-ranked players.

Once a shot is attacked, the success rate of FOS and FOS + 1 is critical to the rally’s outcome, no matter in which shot category the FOS is launched. A similar pattern has also been discovered in men’s matches [4]. Comparing OG16 to OG20 concerning the shot number of FOS, the occurrence rates of FOS^#^3 and FOS^#^≥4 decreased by 1.7% and 3.2%, respectively, while that of FOS^#^2 increased by 5.0%. (OG16: FOS^#^2 = 45.1%, FOS^#^3 = 36.9%, FOS^#^≥4 = 17.9%, OG20: FOS^#^2 = 50.1%, FOS^#^3 = 35.2%, FOS^#^≥4 = 14.7%). One might assume that after the ITTF upgraded the material and size of the ball in 2014, it took players some time to adapt to the features of the new ball, and FOS techniques and tactics gradually advanced.

### Extensions

**Fig. 5:**
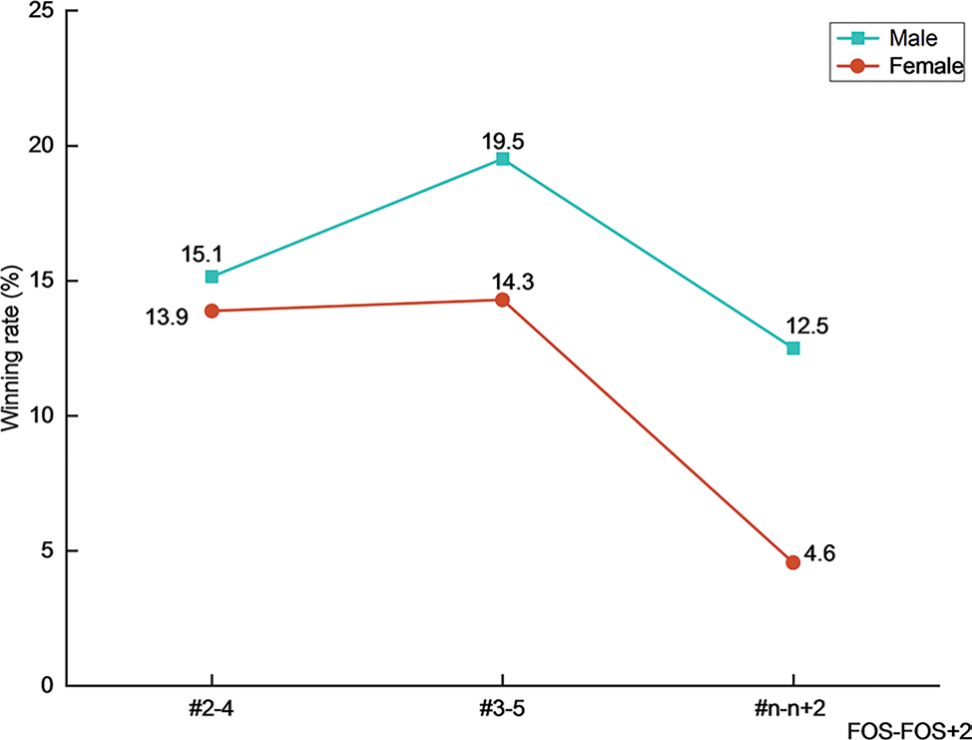
Winning rates of different FOS and FOS + 2 connections of male and female players. Legend: #2–4, #3–5 and # n-*n* + 2 denote the 2nd to 4th shot, 3rd to 5th shot, and n^th^ to (*n* + 2)^th^ (*n* ≥ 4) shot, respectively

Interestingly, male players exhibited greater direct winning rates on FOS^#^2 and FOS^#^≥4, as well as greater overall winning rates on FOS^#^3 (Fig. 3a and b). Moreover, they demonstrated significantly higher winning rates and lower error rates than females on FOS + 2 (Table 5). Table tennis practitioners are frequently interested in two consecutive shots [31], which is often referred as the connecting ability, or tactic, i.e., FOS and FOS + 2. Figure 5 depicts the winning rates of male and female players from FOS to FOS + 2 based on different FOS categories. Male players exhibited higher winning rates in each connection. Some scholars [12, 32] have previously claimed that male players have more winning actions or higher winning rates than female players. This study, for the first time, explored and confirmed that male players achieved higher winning rates than female players in two consecutive initial attacks.

### Limitations and implications

The limitations and practical implications of the study can be examined from three perspectives.

In general terms, when modelling, a researcher has to choose a level of abstraction that is, in a way, arbitrary. In this study, we presented a detailed EFOS model, focusing on investigations of IOP. While we consider this approach a commendable compromise between complexity and validity for table tennis rallies, there still remains room for analysts to explore more detailed models, for instance, differentiating POP and FOP or introducing additional critical aspects such as technique, placement, or footwork [3, 5].

The specific limitations of state-transition models hold in our case, too. Practical interpretations must always consider transition probabilities as well as transition frequencies [33]. In this study, we employed the usage rate as compensation. Researchers can also look for appropriate algorithms to fuse the transition frequencies of each state with the transition probabilities, forming a weighted transition matrix that synthesises the response to a player’s performance. In addition, to identify the causes for weak or strong transition rates, which is a prerequisite for formulating targets for training [1], a detailed qualitative inspection must still be conducted.

Further limitations may result from the underlying sample. First, the study only applies to offensive players, which may introduce bias against defensive players. In light of the technical characteristics of defensive players, (1) their offensive frequency is noticeably lower than that of offensive players, i.e., the utilisation of FOS is very low; (2) it is common for them to transition back to a chopping technique after an offensive shot, i.e., employing defensive techniques once again, which is almost impossible for offensive players. Thus, the exclusion of defensive players is justified to reduce confounding factors arising from fundamental differences in technical and tactical patterns between playing styles and ensure analytical consistency within offensive play dynamics. This may raise another interesting research topic: profiling the transitions in which only defensive players are involved. This may require an additional state transition framework based on the technical system and tactical characteristics of the defensive players. A second limitation of the sample is that it comprises matches from the Olympic Games, which doubtlessly showcase the top level of table tennis. However, the ranking threshold ensured balanced sample sizes between groups, but it may suffer from an overrepresentation of players from various countries, which could lead to differences in match patterns compared to other international tournaments with fewer low-ranked players. As Olympic selection rules limit the number of top-ranked players per entitled member association, this sample distribution could differ from typical world rankings and impact observed performance patterns, as was already mentioned when presenting the results of Yu and Gao [4], who analysed final-stage matches in top-level tournaments. Furthermore, players may endure greater psychological and situational pressures compared to other international tournaments. These factors could affect players’ tactical choices or error tendencies, introducing potential confounding effects.

For coaches and players, the EFOS model highlights at least, but not limited to, three valuable insights: (1) prioritise training on the initial-offensive phase, where over 70% of rallies conclude, focusing on improving both the quality of the FOS and the ability to counterattack offensive shot, i.e., FOS + 1 and FOS + 3; (2) improve shot consistency to reduce direct errors, especially for low-ranked players, as direct errors occurred at a significantly higher rate than direct scores across all states, training programs should focus on maintaining rally stability under pressure, where forced errors are common; (3) develop gender-specific strategies—female players may benefit from refining early attacks (FOS^#^3), while males should enhance counterattacks after defensive returns (FOS^#^≥4).

State-transition models have been successfully applied in various sports performance analysis such as tennis [25], football [34], ultimate frisbee [35]. These models typically segment a rally or game into discrete states based on certain attributes such as technique, tactic, or position, thereby capturing how each state or phase contributes to the outcome. The methodology employed in the EFOS model shares conceptual foundations with these frameworks, yet it also exhibits unique adaptations tailored to the rapid dynamics of table tennis. Specifically, by introducing a new set of performance indicators that combine technical attributes with shot number, this study represents the first integration of table tennis techniques, shot number, and state-transition analysis. Unlike football models focusing on spatial transitions or tennis frameworks emphasising serve dominance, the EFOS model captures the interaction between technical execution and tactical timing, providing a new idea for assessing player performance.

## Conclusion

This study introduces the Enhanced First Offensive Shot (EFOS) model as a novel approach for understanding the dynamics of table tennis rallies. By moving beyond conventional shot-based analysis and adopting a state-transition model, the EFOS model provides a clearer way to capture the interaction between player actions and rally outcomes. The study identifies three key findings. Firstly, the initial-offensive phase emerges as the pivotal phase, where the rally is concluded most frequently, with the first offensive shot and the subsequent shot significantly influencing rally outcomes. Secondly, attacking defensive shots are proven to be more effective than targeting serves. Thirdly, male players outperform female players in both initial and subsequent attacks. These findings provide invaluable practical insights for table tennis players and coaches, suggesting that training should emphasise the initial-offensive phase and focus on reducing errors during transitions, particularly for low-ranked players. Additionally, gender-specific training strategies may be developed to address the observed performance differences. Future research could extend this approach to defensive players in table tennis by constructing a new state system, or to other rapid-transition sports such as badminton or squash, where technical-tactical synergy may predominantly affect match results.

## Data Availability

The datasets used and analysed during the current study are available from the corresponding author on reasonable request.

## Abbreviations

FOS: First Offensive Shot
EFOS: Enhanced First Offensive Shot
POP: Pre-offensive phase
IOP: Initial-offensive phase
FOP: Final-offensive phase
ITTF: International Table Tennis Federation
OG16: Olympic Games Rio 2016
OG20: Olympic Games Tokyo 2020
